# Preparation and Properties of the 3-pentadecyl-phenol In Situ Modified Foamable Phenolic Resin

**DOI:** 10.3390/polym10101124

**Published:** 2018-10-10

**Authors:** Tiejun Ge, Kaihong Tang, Yang Yu, Xiapeng Tan

**Affiliations:** 1 Plastic Engineering Research Center of Shenyang University of Chemical Technology, Shenyang 110142, China; getiejun@syuct.edu.cn (T.G.); lanlefeng@163.com (K.T.); txp351183456@163.com (X.T.); 2 Liaoning Polymer Materials Engineering and Technology Research Center, Shenyang 110142, China; 3 Shenyang Huada and Kangping Plastic Woven Research Institute, Shenyang 110142, China

**Keywords:** 3-pentadecyl-phenol, foamable phenolic resin, in situ modification, toughness

## Abstract

In this present study, 3-pentadecyl-phenol was selected as a modifier to prepare a foamable phenolic resin with excellent performance, which was successfully prepared by in situ modification. Fourier transform infrared spectroscopy (FT-IR) and nuclear magnetic resonance (^1^H NMR, ^13^C NMR) were used to test and characterize the molecular structure of the modified resin. The results showed that 3-pentadecyl-phenol successfully modified the molecular structure of phenolic resin with a reduction in the resin gel time. The effect of changing the added amount of 3-pentadecyl-phenol on the mechanical properties, microstructure, and flame retardancy of the modified foam was investigated. The results showed that when the amount of added 3-pentadecyl-phenol was 15% of the total amount of phenol, this resulted in the best toughness of the modified foam, which could be increased to 300% compared to the bending deflection of the unmodified phenolic foam. The cell structure showed that the modified phenolic foam formed a more regular and dense network structure and the closed cell ratio was high. Furthermore, the compressive strength, bending strength, and limited oxygen index were improved, while the water absorption rate was lowered. However, the foam density could be kept below 40 mg/cm^3^, which does not affect the load.

## 1. Introduction

Phenolic foam is a new generation of flame retardant, thermal insulation, and sound insulation materials [1,2,3]. Its rapid development has resulted in it being widely used in construction, defense, aviation, energy and other fields [4,5] due to its low density, low flammability and low toxicity [6]. However, its shortcomings are also very obvious, including its low elongation, brittleness, high hardness, poor toughness, etc. [7]. Phenolic resin (PF) is the main raw material used in the production of phenolic foam. Its use level is about 50%–90% of the total weight of the phenolic foam [8,9]. The molecular structure of the phenolic resin determines the brittleness of the phenolic foam because there are three active sites on the benzene ring of phenol, which are located on the ortho-position and para-position of the phenolic hydroxyl group. During the polymerization process, polycondensation occurs, which involves these three sites reacting with formaldehyde, and the degree of crosslinking is large. The interior of the resin gradually forms a spatial molecular network with a relatively high cross-link density, with the toughness of the phenolic foam inevitably declining [10]. Thus, if the active point on the benzene ring can be reduced, the toughness of the phenolic foam can be improved.

Shen, H. et al. used fibers to alter the foaming and curing characteristics of phenolic foam [11], while Auad, M.L. et al. used epoxy resin to enhance the mechanical properties of the foam [12]. Zhou, J. et al. used glass fibers [13], while Yang, Y. et al. used short fibers and polyurethane prepolymers to modify the phenolic foams to improve their toughness and strength [14]. Dong, H.J. et al. modified the phenolic resin with aluminum phosphate to prepare a composite material with improved mechanical properties and heat resistance [15]. Wu, C.Z. et al. used polyurethane prepolymer as a toughening agent for the phenolic foam in order to physically toughen the phenolic foam [16]. In terms of the molecular structural characteristics of phenolic resins, Hu, X.M. et al. introduced polyethylene glycol (PEG) into the phenol-urea-formaldehyde foam to improve its toughness [17]. Carvalh, G. et al. [18], Li, J.; Wang, W. et al. [19], Li, J. and Zhang, J. et al. [20] all used a lignin moiety to replace the phenol with formaldehyde to form a phenolic resin to increase its viscosity, reduce formaldehyde emissions, and increase the strength of the phenolic foam. Ma, Y. et al. introduced DOPO-ITA into the phenolic resin to improve the residual carbon and the cell distribution [21]. Turunen, M. et al. used starch, urea and lignin as the phenolic resin modifiers to change the methylene bridge content of the resin by changing the molar mass ratio [22]. Mirski et al. altered the structure using ester modified phenolic resins of different carbon chain lengths and reduced the gel time at 130 °C [23].

According to the above-mentioned studies, the modification of phenolic foam can be divided into two methods [7]. One is the physical modification, which is called the external toughening method. The toughening agent is mixed with a common phenolic resin before being foamed and cured. Another is the chemical modification, which is called the internal toughening method. The modifier is used to modify the molecular structure of the phenolic resin and a flexible chain is inserted into the molecular structure of the phenolic resin. Therefore, in order to comply with current research directions in reducing the active sites on the benzene ring, we chose chemical modification to modify the phenolic resin. Chemical modification is divided into in situ modification and synthetic modification. The in situ modification method involved the modifier with a flexible chain being added directly during the synthesis of the phenolic resin to form a new resin. The synthetic modification method first involves the modification of phenol, before the modified phenol is reacted with formaldehyde to synthesize a new resin. We selected the in situ modification method to toughen the phenolic resin.

Alkylphenols are a class of compounds produced by the alkylation of phenols. Long-chain alkylphenols are widely used in the synthesis of fine chemicals. They are used as additives in fuels, lubricants, and polymers, while alkylphenols are also used as phenolic acids. At the same time, alkylphenol is used as a raw material to create phenolic resin instead of phenol in order to ultimately form a novel resin, which can be used as a binder and a dispersant [24,25,26]. Furthermore, it is also used in thermosetting phenolic resins [27]. Ma, L.Q. et al. used a boron and alkylphenol double-modified phenolic resins to improve their heat resistance [28]. Xiao, X.J. et al. prepared an environmentally friendly resin using nonylphenol and formaldehyde as the raw materials [29]. Geng, X.D. et al. prepared a mixed alkylphenol phenolic resin condensate by means of the condensation of the mixture of p-texylphenol and p-nonylphenol or dodecylphenol with formaldehyde before the condensate was reacted with the molten rosin [30]. Li, P. et al. demonstrated that the molecular chain flexibility of the rosin-modified resin is better as this resulted in a lengthened carbon chain of the alkylphenol substituent [31]. We also used alkylphenol and glutaraldehyde to partially replace phenol and formaldehyde to prepare a composite modified resole phenolic resin, with this obtained phenolic resin having been mixed with the base resin and foamed [32]. However, due to the introduction of glutaraldehyde, the free formaldehyde content of the phenolic resin increased. Although the bending deflection of the foam is increased, the strength is lowered and the density is increased.

The 3-pentadecyl-phenol is a solid with a melting point of approximately 50–53 °C, which is prepared from nut shell oil, and has a fifteen-carbon long chain at the meta position of phenol, which greatly increases its flexibility compared to phenol. Therefore, we chose 3-pentadecyl-phenol alone in this present study to modify the phenolic resin and to reduce the influence of glutaraldehyde on the properties of the phenolic resin and phenolic foam. The effects of 3-pentadecyl-phenol on the properties (such as the resin molecular structure, gel time, free formaldehyde content, solid content, foam density, strength [33], toughness, and cells) of the expandable phenolic resin were investigated.

## 2. Materials and Methods

### 2.1. Materials

Phenol, paraformaldehyde, sodium hydroxide and n-pentane (as a blowing agent) were purchased from the Tianjin Damao Chemical Reagent Factory (Tianjin, China). As the catalyst, sodium hydroxide was dissolved in distilled water to create a saturated solution before use. We obtained 3-pentadecyl-phenol from Tianjin Heowns Biochemical Technology Co., Ltd. (Tianjin, China). The above-mentioned raw materials and reagents were all analytical reagents (≥99.7%). Tween-80 (as a surfactant) and benzenesulfonic (as a curing agent) were supplied by Sinopharm Chemical Reagent Co., Ltd. (Shanghai, China). Both reagents are chemically pure (≥99.5%).

### 2.2. The Synthesis of Modified Phenolic Resin

The modified resin synthesis formula is as follows. The molar ratio of phenol to aldehyde is 1:1.4, while the amount of 3-pentadecyl-phenol is approximately 5%–25% (the step was 2%) of the total amount of phenol. The amount of the basic catalyst was 1% of the total amount of the phenol.

**Scheme 1:**
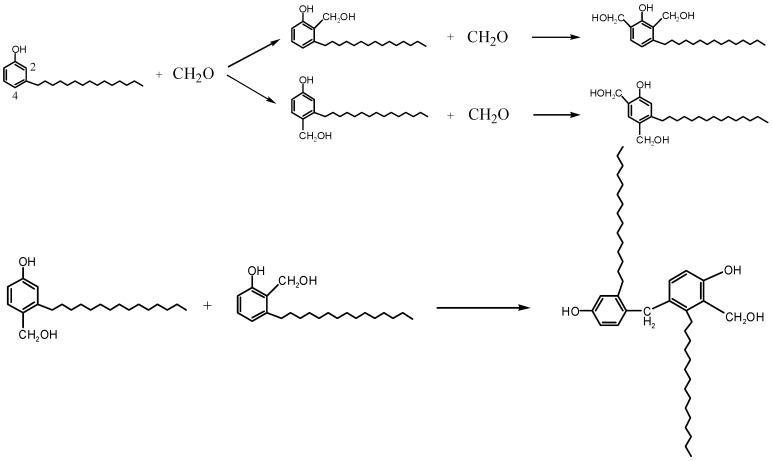
The synthesis mechanism of the phenolic resin modified by 3-pentadecyl-phenol.

The synthetic route for phenolic resin modified by 3-pentadecyl-phenol is shown in Scheme 1, while the detailed synthetic methods are described below.

We first added phenol and 3-pentadecyl-phenol to the three-necked flask (Tianjin Damao Chemical Reagent Factory, Tianjin, China) which was stirred using a paddle according to the ratio (the temperature of the water bath is about 60–65 °C). After this, we added a certain amount of sodium hydroxide as the catalyst and stirred it evenly for about 5 min, which allowed for the activation of phenol and modifiers. After we added the paraformaldehyde into the flask (Tianjin Damao Chemical Reagent Factory, Tianjin, China) in three batches within half an hour, we maintained the temperature of the water bath for 30–40 min and let the paraformaldehyde react fully. After this, we raised the temperature of the water bath to 85–90 °C and allowed the mixture to react continuously for about 90 min to obtain the modified phenolic resin.

### 2.3. Preparation of Phenolic Foam

The phenolic resin foaming formulation is shown in Table 1 (modified phenolic foam and common phenolic foam use the same formulation and conditions). We first sealed the phenolic resin at room temperature for 24 h. After that, we mixed the resin, the surfactant, and the foaming agent well according to the formula shown in Table 1. We subsequently added a certain amount of curing agent, mixed it well, poured the resin into the mold (open glass mold), and placed it in the oven (Beijing ever bright medical treatment instrument co., Ltd., Beijing, China) (the temperature of the oven is about 70–75 °C) for ten minutes to allow it to be cured.

### 2.4. Characterization

The structure of the modified phenolic resin was characterized by an FT-IR (NEXUS 470 Thermo Electron Corporation, Shanghai, China) test with a scanning range of 400–4000 cm^−1^ at a resolution of 4 cm^−1^ and a scanning number of 10. For this, we used an AVANCE III NMR spectrometer with tetramethylsilane (TMS, Tianjin Damao Chemical Reagent Factory, Tianjin, China) as an internal primary standard substance.

The solid content and the free formaldehyde content of the resin were determined according to the Chinese National Standard (GB/T 14074-2006). The resin gel time was determined by taking 1.0 g of resin, pouring it on a hot plate at 150 °C (±1 °C), starting the stopwatch and continuously stirring the resin with a glass rod until the resin was not gelled and stopped.

The bending performance was determined according to the Chinese National Standard (GB/T 8812-2007) with an RGL-type microcomputer control electronic universal testing machine (Shenzhen Rui Geer Instrument Co., Ltd., Shenzhen, China). We used the following parameters: support span of 60 ± 1 mm; indenter arc radius of 5 ± 0.2 mm; sample size of length 100 ± 0.5 mm; width of 10 ± 0.5 mm; and thickness of 4 ± 0.2 mm. The number of samples in each group is five, while the head speed is 3 mm/min. This test records the fracture displacement of the foam, which is called the bending deflection. The compressive strength was determined according to the Chinese National Standard (GB/T 8813-2008).

The foam water absorption rate was determined according to the Chinese National Standard (GB/T 8810-2005). The limiting oxygen index was determined according to the Chinese National Standard (GB/T 2046-1993) with LOI (JF-3 Nanjing Analytical Instrument Factory Co., Ltd., Nanjing, China). The cell structure before and after modification was observed with a scanning electron microscope (EVO10 Carl Zeiss, Oberkochen, Germany). The heat resistance of the foam was analyzed under an air atmosphere using a thermogravimetric analyzer (STA 449C NETZSCH-Gerätebau, Selb, Germany) at a temperature of 40–800 °C and a heating rate of 10 °C/min. The sample quantity is around 6 mg.

## 3. Results and Discussion

### 3.1. Resin Structure

#### 3.1.1. FT-IR Analysis

**Figure 1:**
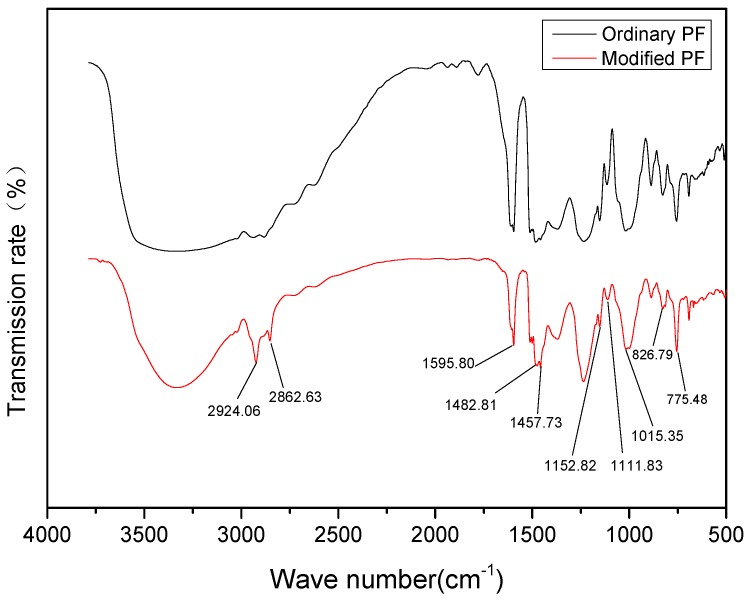
The FT-IR spectra of the ordinary PF and modified PF (the amount of 3-pentadecyl-phenol is 15% of the total amount of phenol).

Figure 1 shows the FT-IR spectra of the ordinary phenolic resin and the 3-pentadecyl-phenol modified phenolic resin. The stretching vibration region of the benzene ring skeleton is located at 1620–1430 cm^−1^, while the IR spectrum has a strong absorption peak at about 1595.8–1482.81 cm^−1^. There are absorption peaks at 755.48 cm^−1^ in the ortho-substitution bending vibration region of the benzene ring and 826.79 cm^−1^ in the para-substitution bending vibration region of the benzene ring. Compared with the ordinary phenolic resin, the IR spectra of 3-pentadecyl-phenol modified phenolic resin showed the bands at 2924.09 and 2862.63 cm^−1^, which are characteristic of the –CH_2_– groups in the pentadecyl long chain. An IR band arising from the alkyl bending vibration was observed at 1457.73 cm^−1^. The IR bands of the C–O group of phenol and C–O group of hydroxymethyl appeared at 1152.82, 1015.35, and 1111.83 cm^−1^, respectively.

In the infrared spectrum of the 3-pentadecyl-phenol modified phenolic resin, the ratio of the stretching vibration peak at 1015.35 cm^−1^ to the stretching vibration peak at 1152.82 cm^−1^ is significantly larger than the ratio of these two peaks of the unmodified phenolic resin. In essence, in the 3-pentadecyl-phenol modified phenolic resin, the hydroxymethyl content of the C–O group is higher than that of the ordinary phenolic resin. The reason for this phenomenon is that the 3-pentadecyl-phenol partially replaced phenol. The main structure of 3-pentadecyl-phenol includes a long chain with a 15-carbon at the meta position of phenol and active sites in its ortho- and para-positions. When synthesizing the phenolic resin, the active site on the ortho-position is separated by the alkyl group on the benzene ring and the benzene hydroxyl group so that the hydroxymethyl at position 2 (Scheme 1) in the molecular structure of 3-pentadecyl-phenol modified phenolic resin is not polycondensed with formaldehyde.

The 3-pentadecyl-phenol and formaldehyde are first subjected to an addition reaction under alkaline conditions before the hydroxymethyl group is randomly connected to the ortho-position or para-position of the 3-pentadecyl-phenol. After this, they continue to undergo an addition reaction with formaldehyde to complement these positions. When the free paraformaldehyde is completely reacted with 3-pentadecyl-phenol, the temperature is raised to cause a polycondensation reaction. Because the long carbon chain in the 3-pentadecyl-phenol meta-position increases the steric hindrance of the system and separates the active sites on the ortho-position, the polycondensation reaction takes place preferentially on position 4 in the molecular structure of 3-pentadecyl-phenol modified phenolic resin, which produces a phenolic resin as described in the above-mentioned reaction formula (Scheme 1).

#### 3.1.2. ^1^H NMR Analysis

**Figure 2:**
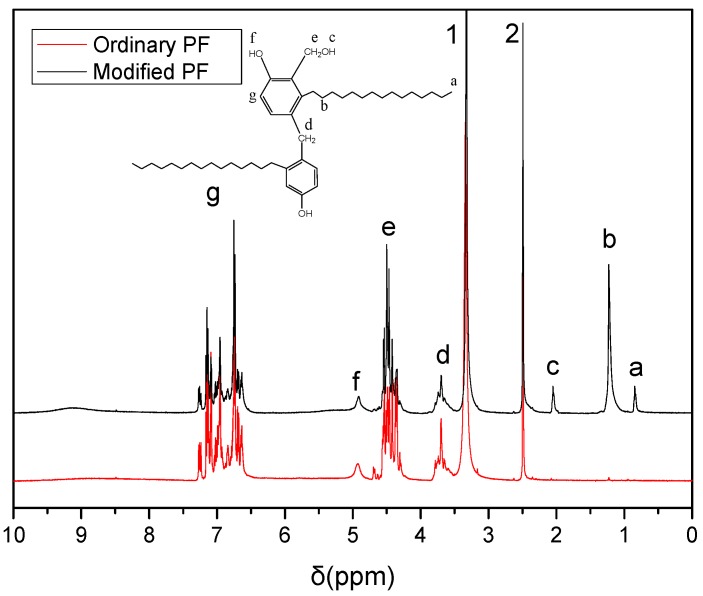
The ^1^H NMR spectra of the ordinary PF and modified PF (the amount of 3-pentadecyl-phenol is 15% of the total amount of phenol).

Figure 2 shows the ^1^H NMR spectra of the 3-pentadecyl-phenol modified phenolic resin and ordinary phenolic resin. It can be seen from Figure 2 that δ = 6.5–7.3 ppm at position g is the proton peak of hydrogen in the benzene ring, while δ = 4.8–5.1 ppm at position f is the proton peak of hydrogen in phenol, while δ = 4.15–4.65 ppm at position e is the hydrogen proton peak of the methyl group in the hydroxymethyl group. At the position d, δ = 3.5–3.9 ppm is the proton peak of the hydrogen in the linking benzene ring –CH_2_–. Furthermore, position c has δ = 1.9–2.1 ppm, which is the hydrogen proton peak of hydroxymethyl –OH, while the δ = 0.8–1.4 ppm at positions a and b are the proton peaks of the hydrogen in the fifteen carbon long chain. The δ = 3.3 ppm at position 1 is the proton chemical shift of H_2_O involved in NMR, while the δ = 2.5 ppm at position 2 is the chemical shift of the deuterated solvent DMSO.

From the ^1^H NMR spectrum, it can be seen that the expected chemical structure of the 3-pentadecyl-phenol modified phenolic resin is formed. Since the area of the proton peak in the ^1^H NMR spectrum is proportional to the amount of hydrogen, it can be obviously seen that the content of methylene –CH_2_– at the δ = 3.5–3.9 ppm in the modified resin is lower than that of the ordinary resin, while the content of hydroxymethyl groups is more than that of ordinary resin. Looking at the IR spectrum, the active site in the ortho-position 2 is separated by the phenolic hydroxyl group and the 15-carbon long chain in the 3-pentadecyl-phenol so that the hydroxymethyl at position 2 in the molecular structure of 3-pentadecyl-phenol modified phenolic resin is not polycondensed with formaldehyde to form methylene bridges. The content of methylene groups linked to the benzene ring in the modified phenolic resin decreases, while the content of hydroxymethyl groups increases. Due to the decrease in the cross-linking density of phenolic hydroxyl groups in the ortho-position, the cross-linking density of the para-position will increase, making the arrangement of the modified phenolic resin space network more regular and increasing the strength of the phenolic foam [10].

#### 3.1.3. ^13^C NMR Analysis

**Figure 3:**
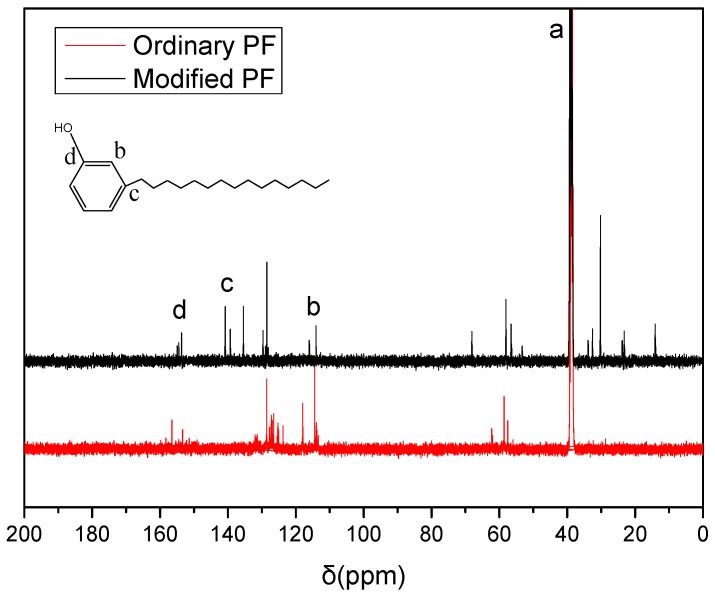
The ^13^C NMR spectra of the ordinary PF and modified PF (the amount of 3-pentadecyl-phenol is 15% of the total amount of phenol).

Figure 3 shows the ^13^C NMR spectrum of the 3-pentadecyl-phenol modified phenolic resin and ordinary phenolic resin. It can be seen that δ = 153.2–154.7 ppm at position d is the peak of the carbon on the benzene ring in the phenolic hydroxyl group; δ = 113.3–141.5 ppm are the peaks of other carbons on the benzene ring except the phenolic hydroxyl group; δ = 52.7–69.2 ppm is the peak of the carbon in the hydroxymethyl group; and δ = 14.1–33.1 ppm is the peak of the fifteen carbon long chain in 3-pentadecyl-phenol. The peak at δ = 29.6 ppm is the peak of the methylene group attached to the benzene ring. The δ = 39.3–40.6 ppm at the position a is the chemical shift of the deuterated solvent DMSO.

The peaks of carbon in the phenol benzene ring without the fifteen carbon long chain are relatively uniform. Looking at the ^13^C NMR spectra of the phenolic hydroxyl group at the two ortho-position carbon proton peaks and two para-position carbon proton peaks, the chemical shifts are similar or even the same. This is because the electron cloud density is relatively uniform and the molecular structure is stable. The peak shift interval of the carbon in the phenol benzene ring that is connected to the fifteen carbon long chain is obviously larger. In position c, approximately δ = 139.2 ppm is the peak of the carbon at the benzene ring that is connected with the fifteen carbon long chain. The peak of the carbon atoms which are substituted generally shifts towards the low field, which results in a higher density of electrons in the intermediate carbon of the fifteen carbon chain and the phenolic hydroxyl group. Furthermore, the chemical shift is reduced to about δ = 115.3 ppm at position b. Therefore, after the phenol is connected to the fifteen carbon long chain, the density of the electron cloud at each position on the benzene ring is not uniform, the distribution of the electron cloud changes and the intermolecular force of the benzene ring changes. Thus, the modified phenolic foam has a reduced rigidity and improved toughness.

### 3.2. Basic Properties of Resin

As shown in Table 2, the gel time of ordinary phenolic resin is 218 s and the modified phenolic resin gel time can be as short as 175 s. This is because the three active sites on the benzene ring (two ortho-positions and one para-position) can theoretically undergo a methylolation reaction. Increasing the amount of 3-pentadecyl-phenol will increase the proportion of active sites occupied, while the gel time after the modification will be shortened. However, when the methylol content reaches a certain value, the rate of polymerization is greater than the rate of the methylolation reaction and thus, the methylol content in the resin is reduced. As a result, the gel time is first reduced rapidly, before having a slower reduction. The phenolic to aldehyde molar ratio of ordinary unmodified phenolic resin is 1:1.5–2.0, while the molar ratio of phenol to aldehyde is 1:1.4 in this experiment so the reaction is more complete and the free formaldehyde content is reduced [34]. However, the 3-pentadecyl-phenol is a solid and as the content of 3-pentadecyl-phenol increases, the viscosity of the reaction solution increases. In order to maintain the viscosity of the synthesized resin in the same range, the temperature and reaction time are appropriately adjusted as the 3-pentadecyl-phenol increases [35]. The reaction temperature and the reaction time are reduced according to the amount of 3-pentadecyl-phenol so that the reaction is not as complete as before and the content of free formaldehyde is increased. The content of 3-pentadecyl-phenol does not affect the solid content of the resin.

### 3.3. Mechanical Properties of the Foam

#### 3.3.1. Bending Performance Analysis

**Figure 4:**
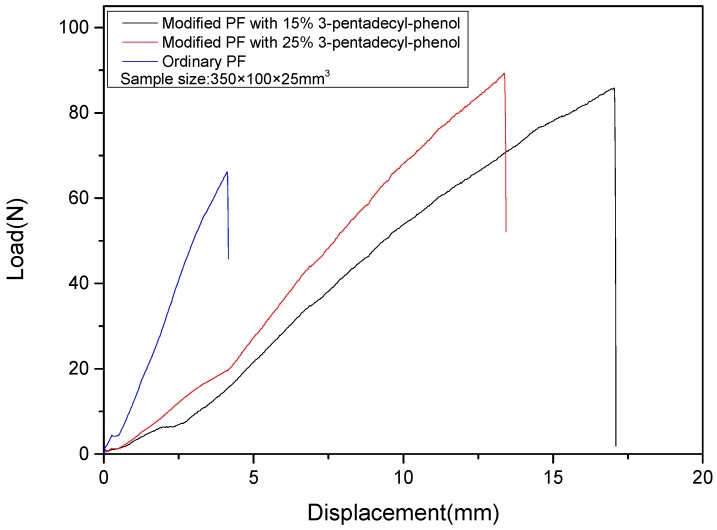
The bending characteristics of the phenolic foam: load-displacement curves.

As shown in Figure 4, the slope of the foam load-displacement curve is decreased as the addition of 3-pentadecyl-phenol is increased. According to Hooke’s law, the smaller the slope of the foam load-displacement curve, the greater the elastic modulus is and the foam toughness is the better [33]. As the amount of added 3-pentadecyl-phenol is too large, the foam bending strength increases, but the slope of the foam load-displacement curve begins to increase and the toughness decreases.

**Figure 5:**
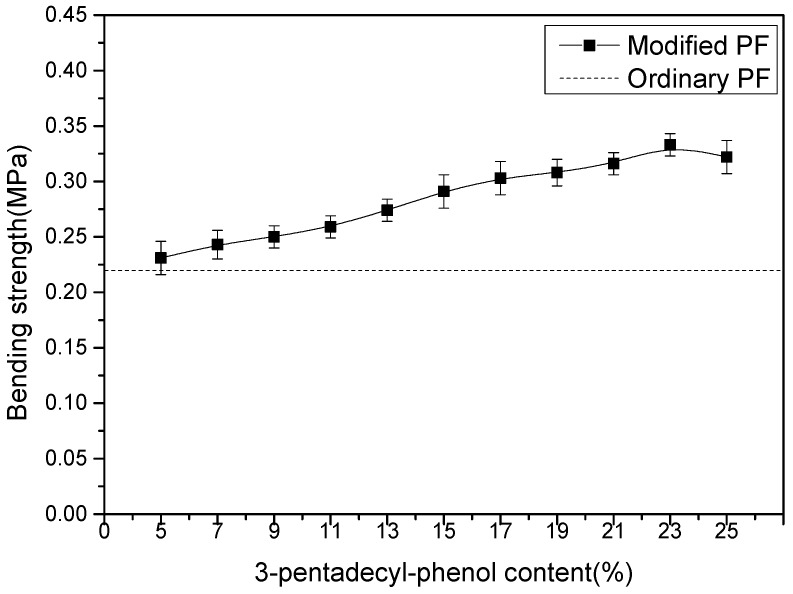
The effect of 3-pentadecyl-phenol with different contents on the bending strength of the foam.

As shown in Figure 5, the bending strength of the ordinary phenolic foam was 0.22 MPa. As the amount of added 3-pentadecyl-phenol increases, the bending strength of the sample gradually increases to 0.32 MPa. This is because, with the addition of 3-pentadecyl-phenol, the space molecular network of the phenolic resin is improved, the arrangement is more regular and the strength of the phenolic foam is improved [36]. The increase in the strength of the phenolic foam increases the maximum load that is required to bend the sample and thus when the amount of 3-pentadecyl-phenol is gradually increased, the bending strength of the modified phenolic foam increases. However, when the amount of added 3-pentadecyl-phenol is too large, the denseness of the foam-molecular cross-linking network structure is reduced and the strength of the inter-molecular connection is insufficient, resulting in a decrease in the bending strength.

**Figure 6:**
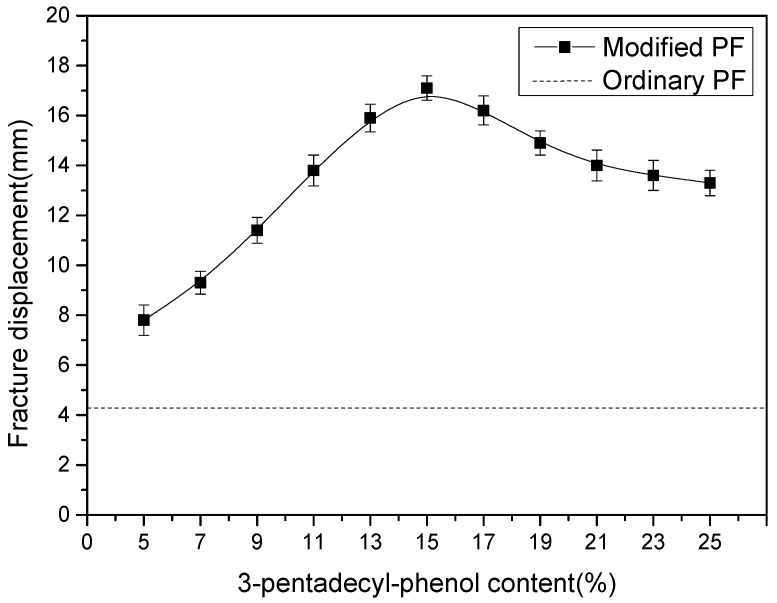
The effect of 3-pentadecyl-phenol with different contents on the bending deflection of the foam.

As shown in Figure 6, the fracture displacement of ordinary phenolic foam was only 4.3 mm and the addition of 3-pentadecyl-phenol greatly increased the fracture displacement of the modified phenolic foam. This is because the introduction of fifteen carbon long chains reduces the rigidity of the benzene ring and the modified phenolic resin space molecular network arrangement becomes more regular, which increases the flexibility of the modified phenolic foam. When the amount of added 3-pentadecyl-phenol was 15%, the bending deflection of the sample was the best and the fracture displacement was 17.1 mm, which was increased by 300% compared with the bending deflection of the ordinary phenolic foam. When the added amount exceeds 15%, the bending deflection slightly decreases. With the addition of 3-pentadecyl-phenol, the viscosity of the modified phenolic resin system increases, the strength of the phenolic foam wall increases, the brittleness increases and the toughness decreases, although it is still 200% higher than normal phenolic resin.

#### 3.3.2. Compressive Strength Analysis

**Figure 7:**
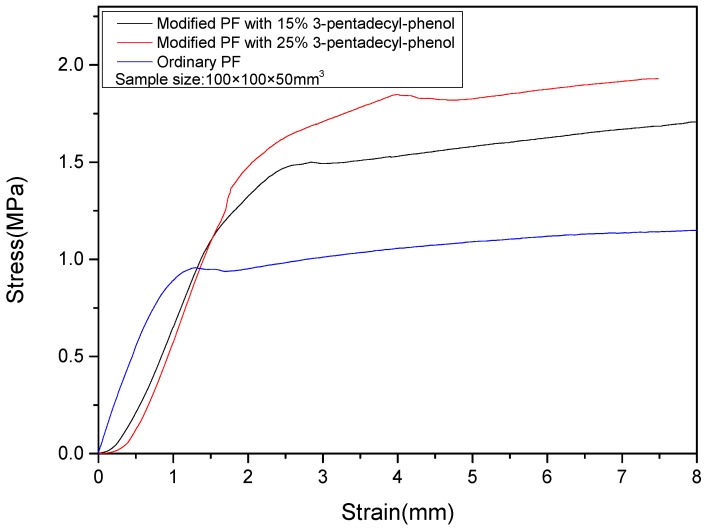
The compressive characteristics of phenolic foam: stress-strain curves.

As shown in Figure 7, the phenolic foam modified by 3-pentadecyl-phenol has a smaller slope of the foam stress-strain curve compared to the unmodified phenolic foam. It can be seen that the modified foam has an increased elastic modulus and a decrease in foam rigidity [33]. With the addition of 3-pentadecyl-phenol, the compressive strength of the foam gradually increases, while the elastic modulus appears to decrease slightly.

**Figure 8:**
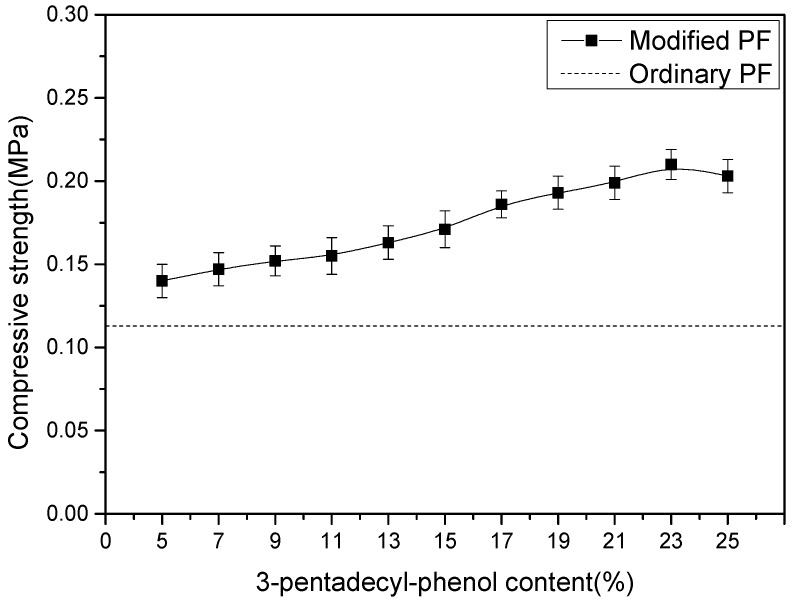
The effect of 3-pentadecyl-phenol with different contents on the compressive strength of the foam.

As shown in Figure 8, the modified phenolic foam has a higher compressive strength compared to the unmodified phenolic foam and the maximum compressive strength is 0.210 MPa when the content of the 3-pentadecyl-phenol is 23% of the total amount of the phenol.

The 3-pentadecyl-phenol increases the flexibility of the modified phenolic foam; the space molecular network of the phenolic resin becomes more regular; the strength of the phenolic foam increases and the maximum load that the foam wall can withstand increases. Thus, the phenolic foam has a higher compressive strength than the unmodified phenolic foam [37,38]. However, as the amount of added 3-pentadecyl-phenol is too large, the density of the interlinked network structure of the modified phenolic foam decreases and foam cells begin to open, which ultimately decreases its compressive strength.

### 3.4. Foam Cell Structure

#### 3.4.1. Apparent Density Analysis

**Figure 9:**
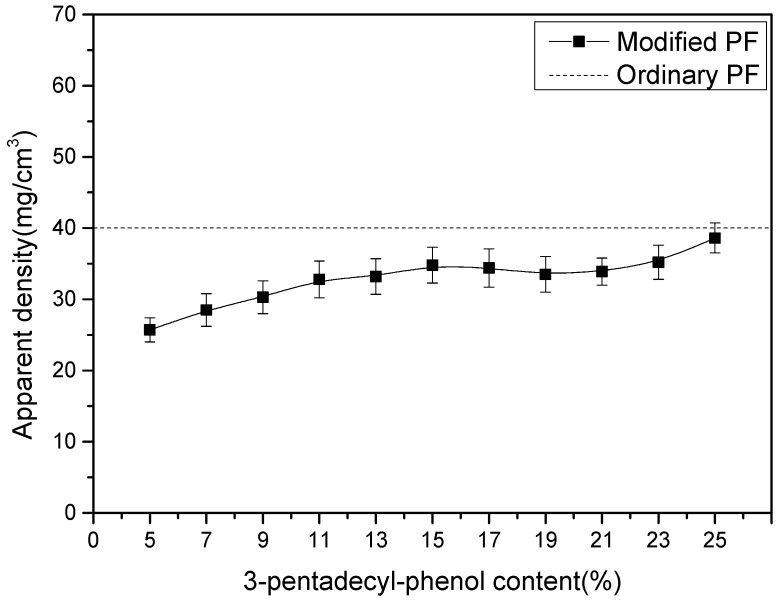
The effect of 3-pentadecyl-phenol with different contents on the apparent density of the foam.

The density of the ordinary phenolic foam is in the range of 40–50 mg/cm^3^ and the modified phenolic foam density is below 40 mg/cm^3^ as shown in Figure 9. Overall, the average density is reduced.

As 3-pentadecyl-phenol increases, the toughness of the phenolic foam, the toughness of the foam wall becomes better and the cells are not easily broken after the addition of the foaming agent. Due to this, the expansion ratio of the foam increases and the foam density decreases [39]. Moreover, the maximum residual pH of the modified foam in the Supplementary Materials Figure S1 is 6.2, so the modified foam as a flame retardant material does not affect its application for production and life. With the addition of 3-pentadecyl-phenol, the density of the modified phenolic foams gradually increases. Because the modifier is solid, a greater amount of added 3-pentadecyl-phenol increases the viscosity of the system, which could affect the foam expansion and results in a greater foam density.

#### 3.4.2. Analysis of Water Absorption Rate

**Figure 10:**
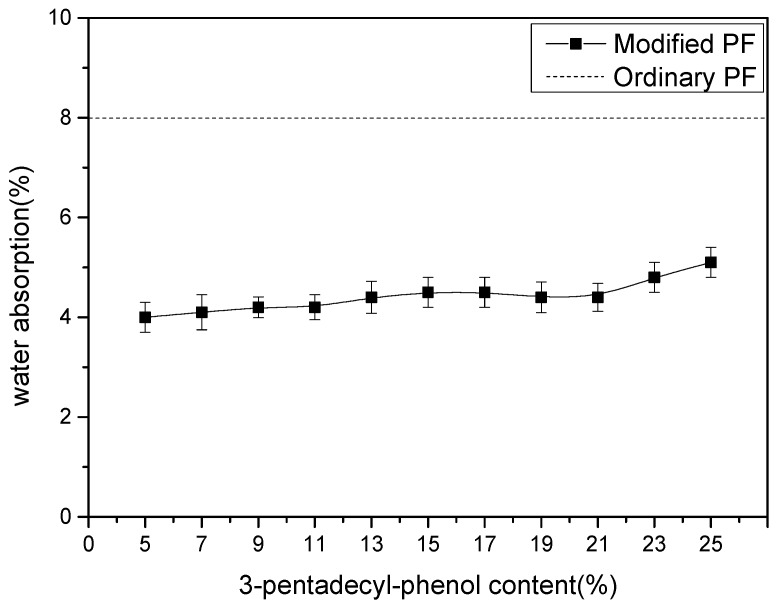
The effect of 3-pentadecyl-phenol with different contents on the water absorption rate of the foam.

In this paper, the water absorption rate is used to judge the opening and closing porosity of phenolic foam cells. As shown in Figure 10, the water absorption of the unmodified phenolic foam is about 8%, while the range of the water absorption of the modified phenolic foam is 4%–5%.

This is because the addition of 3-pentadecyl-phenol increases the toughness of the phenolic foam, improves the toughness of the foam wall, and reduces the bursting of the foaming agent into the cell, which increases the cell closing [40]. As shown in Figure 8, when the addition of 3-pentadecyl-phenol is less than 20%, the effect on the water absorption of phenolic foam is not obvious. However, after 20%, the water absorption of phenolic foam is obviously improved. The viscosity of the resin is increased with the addition of 3-pentadecyl-phenol and the density of the crosslinked network structure of the modified phenolic foam is decreased. The foam has an open cell so the water absorption rate is improved but it is still significantly lower than the ordinary phenolic foam.

#### 3.4.3. Cell Microstructure

**Figure 11:**
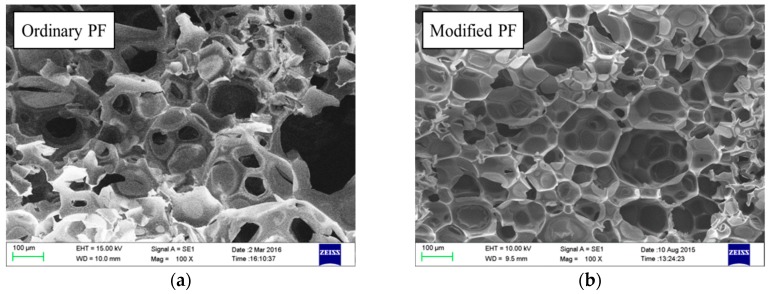
The SEM of PF (**a**) Ordinary PF; and (**b**) Modified PF with the amount of 3-pentadecyl-phenol being 15% of the total amount of phenol.

Figure 11 shows the cell microscope of the unmodified phenolic foam and modified phenolic foam.

The SEM of the ordinary foam shows that the cells of the phenolic foam are not uniform and the opening ratio of the foam is relatively high. Furthermore, looking at the cross-section of the cell, we determined that the gap between cells is not dense enough. The SEM of the modified foam shows that the cell structure of the foam is three-dimensional. The network structure of the modified foam is denser than that of the ordinary phenolic foam. There is almost no gap between the cells. Moreover, the modified phenolic foam has a lower thermal conductivity compared to the ordinary phenolic foam and the minimum thermal conductivity is 0.024 W/(m·K) when the content of the 3-pentadecylphenol is 15% of the total amount of the phenol, as shown in Supplementary Materials Figure S2. The closed cell ratio is high. The foam cells and the thickness of the cell wall are uniform. By comparing the microstructure of the cells, it is shown that the phenolic foam modified by the 3-pentadecyl-phenol has a better performance. The strength of the phenolic foam is increased due to the dense network structure. The high closed cell ratio decreases the water absorption of the phenolic foam. The toughness of the phenolic foam is increased due to the uniform foam cells and cell wall thickness [17,41].

### 3.5. Foam Heat Resistance

#### 3.5.1. Limited Oxygen Index

**Figure 12:**
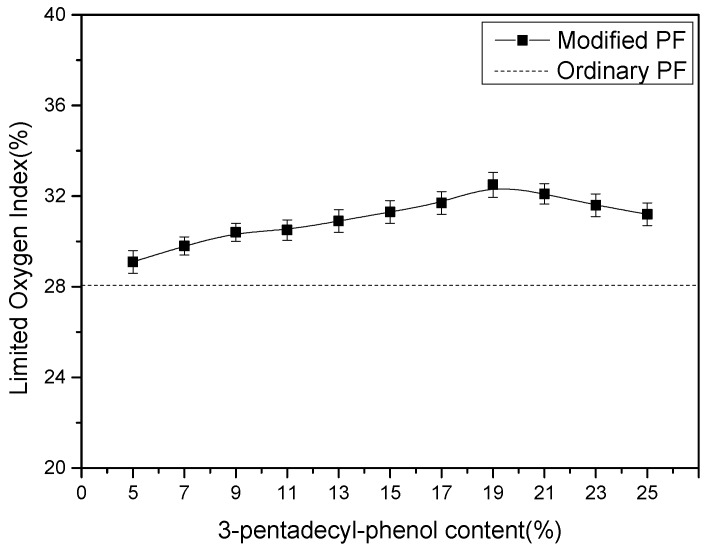
The effect of 3-pentadecyl-phenol with different contents on the limited oxygen index of the foam.

The phenolic resin contains a large amount of aryl groups in the main chain and the pyrolysis residual amount (CR) is high. When burning, the carbonized layer rapidly covers the surface of the burning polymer, thereby extinguishing the flame [42,43]. Figure 12 shows that the flame retardancy of the modified phenolic foam increases slightly with the addition of 3-pentadecyl-phenol. As the 3-pentadecyl-phenol promotes the reaction of phenol and its active sites with formaldehyde in the synthesis stage, the molar ratio of phenol to aldehyde in the experiment is less than that of ordinary phenolic resin so that the content of free formaldehyde in the phenolic foam is reduced. Formaldehyde is flammable and the reduction of the free formaldehyde content causes the flame retardancy of the modified phenolic foam to increase slightly. However, as the amount of added 3-pentadecyl-phenol increases, the viscosity of the resin becomes larger and the formaldehyde does not react completely. Thus, it leads to an increase in the free formaldehyde content and the foam has an open cell. As a result, the flame retardant performance is slightly reduced so the oxygen index decreases.

#### 3.5.2. Thermogravimetric Analysis

**Figure 13:**
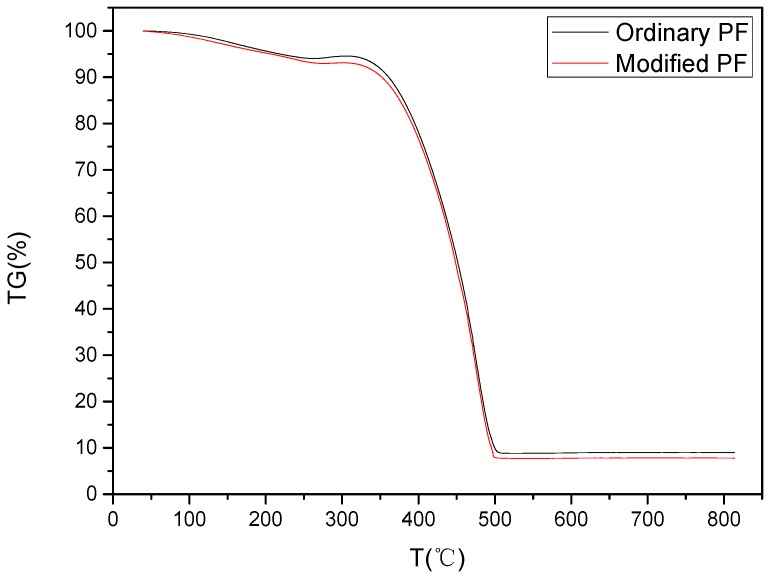
The TG of the ordinary PF and modified PF (the amount of 3-pentadecyl-phenol is 15% of the total amount of phenol).

At about 200 °C, the modified phenolic foam and the unmodified phenolic foam began to decompose, which was mainly due to the loss of free formaldehyde in the foam [44]. At about 400 °C, the phenolic foam begins to partially decompose so the thermal weight loss is accelerated and the rate of weight loss of the two curves is basically the same as shown in Figure 13. The relevant parameters of foam, such as the residual mass and maximum decomposition temperature (*T*_max_), are listed in Table 3. The addition of 3-pentadecyl-phenol does not substantially affect the thermostability of the foam.

## 4. Conclusions

In this paper, 3-pentadecyl-phenol was selected as a modifier to prepare a foamable phenolic resin. The foamable phenolic resin with excellent performance was successfully prepared. When the amount of added 3-pentadecyl-phenol is 15% of the total amount of phenol, the toughness of the modified foam is the best. The bending deflection can be increased by 300% compared to the unmodified phenolic foam. The cell structure shows that the modified phenolic foam has a more regular and dense network structure. The closed cell ratio of the modified phenolic foam is high. The foam cells and cell wall thickness of the modified phenolic foam are uniform. The compressive strength of the foam modified by 3-pentadecyl-phenol reaches a maximum of 0.210 MPa and the bending strength can reach up to 0.32 MPa. The limited oxygen index is improved and the water absorption rate is lowered. However, the foam density can be kept below 40 mg/cm^3^ and does not affect the load.

## Figures and Tables

**Table 1:** The phenolic resin foaming formula.

Phenolic resin (phr)	Foaming agent (phr)	Surfactant (phr)	Curing agent (phr)
100	8	8	12

**Table 2:** The basic properties of the resin.

The content of 3-pentadecyl-phenol of total phenol (%)	Gel time (s)	Free formaldehyde content (%)	Solid content (%)
0	218	0.22	87.6
5	221	0.20	87.9
7	208	0.18	88.0
9	197	0.17	88.4
11	184	0.17	88.6
13	181	0.15	89.1
15	182	0.16	88.8
17	180	0.17	88.1
19	179	0.21	87.7
21	181	0.24	87.0
23	177	0.27	86.4
25	175	0.28	86.3

**Table 3:** The thermal gravimetric of the ordinary PF and modified PF (the amount of 3-pentadecyl-phenol is 15% of the total amount of phenol).

Parameter	PF	Modified PF
*T*_max_ (°C)	503.7	498.5
Residual mass (%)	9.09	8.0

## Supplementary Materials

The following are available online at http://www.mdpi.com/2073-4360/10/10/1124/s1, Figure S1: Effect of 3-pentadecyl-phenol with different contents on the residual pH of foam, Figure S2: Effect of 3-pentadecyl-phenol with different contents on the thermal conductivity of foam.
