# Supplementary material for: Preparation and Properties of the 3-pentadecyl-phenol In Situ Modified Foamable Phenolic Resin
Source: Polymers (Basel). 2018 Oct 10;10(10):1124. doi: 10.3390/polym10101124 (PMC6403807; doi:10.3390/polym10101124)
Supplement: Supplementary file 1 [file polymers-10-01124-s001.pdf]

Foam residual pH

As shown in Figure S1, the modified phenolic foam has a higher residual pH compared to the unmodified phenolic foam and the maximum residual pH is 6.2 when the content of the 3-pentadecyl-phenol is 17% of the total amount of the phenol. When the 3-pentadecyl-phenol added amount exceeds 11%, the foam residual pH is greater than 5. Thus, the modified foam as a flame retardant material does not affect its application for production and life.

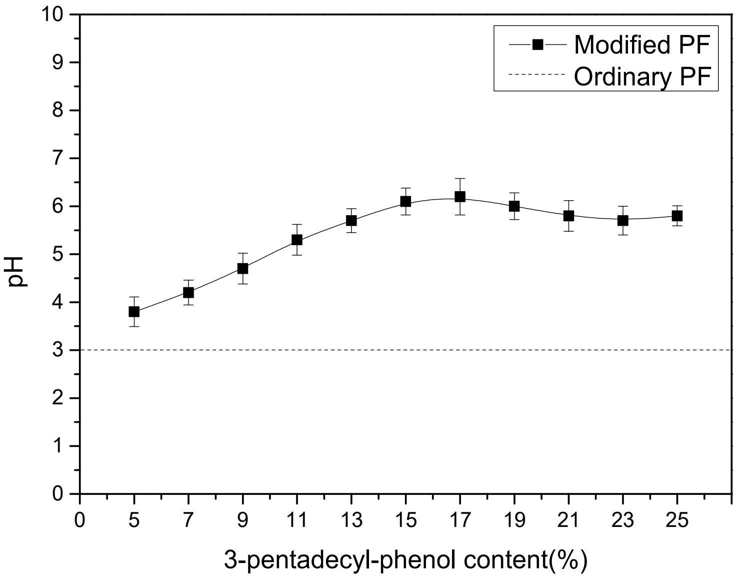

Figure S1. Effect of 3-pentadecyl-phenol with different contents on the residual pH of foam

Thermal conductivity

As shown in Figure S2, the thermal conductivity of the modified phenolic foam dropped first and then increased. The modified phenolic foam has a lower thermal conductivity compared to the ordinary phenolic foam and the minimum thermal conductivity is 0.024 W/(m·K) when the content of the 3-pentadecyl-phenol is 15% of the total amount of the phenol.

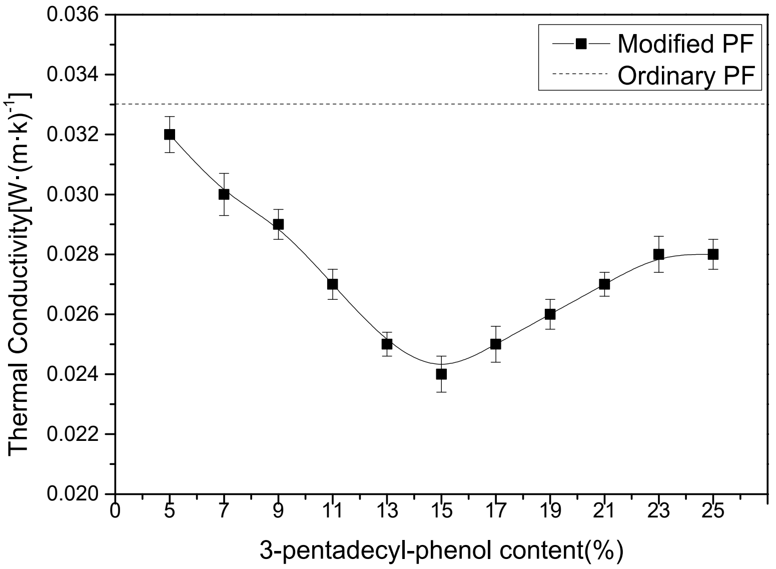

Figure S2. Effect of 3-pentadecyl-phenol with different contents on the thermal conductivity of foam
